# Supplementary material for: Cloud BioLinux: pre-configured and on-demand bioinformatics computing for the genomics community
Source: BMC Bioinformatics. 2012 Mar 19;13:42. doi: 10.1186/1471-2105-13-42 (PMC3372431; doi:10.1186/1471-2105-13-42)
Supplement: Additional file 1 — Supplementary 1 Cloud BioLinux software documentation in the form of a mini, self-contained website. Users need to download and uncompress the .zip file, and open through a web browser the "index.html" file available on the main directory. (ZIP 1823 kb). [file 1471-2105-13-42-S1.ZIP › Cloud-BioLinux-Package-Documentation/docs/primer3.html]

Bio-Linux Software Documentation Pages

Back to search form

## primer3

|  |  |
| --- | --- |
| Name | primer3 |
| Description | **primer3** picks primers for PCR reactions, considering as criteria:-  - oligonucleotide melting temperature, size, GC content, and primer-dimer possibilities. - PCR product size - positional constraints within the source sequence - miscellaneous other constraints.  All of these criteria are user-specifiable as constraints, and some are specifiable as terms in an objective function that characterizes an optimal primer pair. |
| Homepage | http://primer3.sourceforge.net/ |
| Remote Documentation | http://primer3.wiki.sourceforge.net/ |
